# Extracellular vesicle-shuttled miRNAs: a critical appraisal of their potential as nano-diagnostics and nano-therapeutics in type 2 diabetes mellitus and its cardiovascular complications

**DOI:** 10.7150/thno.51605

**Published:** 2021-01-01

**Authors:** Francesco Prattichizzo, Giulia Matacchione, Angelica Giuliani, Jacopo Sabbatinelli, Fabiola Olivieri, Paola de Candia, Valeria De Nigris, Antonio Ceriello

**Affiliations:** 1 IRCCS MultiMedica, Milan, Italy.; 2 Department of Clinical and Molecular Sciences (DISCLIMO), Università Politecnica delle Marche, Ancona, Italy.; 3 Center of Clinical Pathology and Innovative Therapy, IRCCS INRCA, Ancona, Italy.; 4 Institut d'Investigacions Biomèdiques August Pi i Sunyer (IDIBAPS), Barcelona, Spain.

**Keywords:** extracellular vesicles, miRNAs, type 2 diabetes, insulin resistance, β-cell dysfunction, low-grade inflammation, vascular complications, cardiovascular diseases, residual vascular risk, biomarkers, exosomes, microvesicles, diet, exercise, food

## Abstract

Type 2 diabetes mellitus (T2DM) is a complex multifactorial disease causing the development of a large range of cardiovascular (CV) complications. Lifestyle changes and pharmacological therapies only partially halt T2DM progression, and existing drugs are unable to completely suppress the increased CV risk of T2DM patients. Extracellular vesicles (EV)s are membrane-coated nanoparticles released by virtually all living cells and are emerging as novel mediators of T2DM and its CV complications. As a matter of fact, several preclinical models suggest a key involvement of EVs in the initiation and/or progression of insulin resistance, β-cell dysfunction, diabetic dyslipidaemia, atherosclerosis, and other T2DM complications. In addition, preliminary findings also suggest that EV-associated molecular cargo, and in particular the miRNA repertoire, may provide with useful diagnostic and/or prognostic information for the management of T2DM. Here, we review the latest findings showing that EV biology is altered during the entire trajectory of T2DM, *i.e.* from diagnosis to development of CV complications. We also critically highlight the potential of this emerging research field, by describing both preclinical and clinical observations, and the limitations that must be overcome to translate the preclinical findings into the development of EV-based nano-diagnostic and/or nano-therapeutic tools. Finally, we summarize how two lifestyle changes known to prevent or limit T2DM, *i.e.* diet and exercise, affect EV number and composition, with a focus on the possible role of EVs contained in food in shaping metabolic responses, a promising approach still in its infancy.

## Introduction

Type 2 diabetes mellitus (T2DM) is a complex, multi-factorial disease caused by the detrimental interaction between the individual genetic background and a large range of environmental stimuli. T2DM is defined based on the mere presence of chronic hyperglycaemia but a plethora of additional biochemical, molecular, and cellular imbalances have been described in patients with frank T2DM 1,2,3. Several different organs are involved in T2DM etiopathogenesis: alterations affecting the intestine/incretin system, α-cells, the kidney, and specific areas of the brain are emerging drivers beyond the more-studied imbalance of β-cells, adipose tissue, skeletal muscle, and liver 4,5. As a result, patients with T2DM display a large heterogeneity of tangible clinical variables, with different patients showing a variable degree of insulin-resistance, β-cell failure and dyslipidaemia 1. To disentangle this complexity, recent research has also tried to re-categorize patients with T2DM through the dosage of specific clinical variables able to identify the prominent underlying pathological process. In particular, six variables (glutamate decarboxylase antibodies, age at diagnosis, BMI, HbA1c, and homoeostatic model assessment 2 estimates of β-cell function and insulin resistance) are able to distinguish five replicable clusters of patients with diabetes, four of which are commonly considered T2DM 6. Of note, patients in different clusters usually follow different trajectories of complication development: as an example, patients characterized by high insulin resistance show significantly higher risk of diabetic kidney disease while patients characterized by insulin-deficiency most likely suffer from retinopathy 6.

This complex picture also applies to T2DM complications. Despite the continuous advancement of patients' management, T2DM-dependent cardiovascular (CV) complications still represent a major issue. The Steno-2 trial and updated meta-analyses reported that even the treatment with a multi-pharmacological approach aimed at reducing multiple CV risk factors, *e.g.* blood pressure, LDL-cholesterol, and HbA1c is not able to completely suppress the incidence of CV diseases (CVD) in T2DM patients 7, 8. Compared to the general population, the incidence of events relative to heart failure is higher also in T2DM patients with no CV risk factors, 9. Furthermore, despite the preventive role of an early and intensive glycaemic control, the burden of microvascular complications, *e.g.* nephropathy, retinopathy, and neuropathy, negatively impacts the patients' quality of life and represents a major economic cost for the health care system 10,11. All these observations suggest a possible residual vascular (both micro- and macro-) risk in patients with T2DM and imply that additional mechanisms beyond conventional risk factors might contribute to the development of complications in these patients.

Among the wide range of alterations described in T2DM, extracellular vesicles (EV)s are gaining considerable attention. EVs are membrane-coated nanoparticles actively released by virtually all cell types and are commonly classified according to their size and biogenesis 12. Small vesicles of a diameter <100 nm deriving from multivesicular bodies are usually referred to as exosomes. On the other hand, larger EVs (ranging from 200 nm to 1 micron) most commonly derive from direct plasma membrane shedding 13. However, the size of exosomes and microvesicles are now know to largely overlap and thus size *per se* does not rigidly indicate EV biogenesis. Indeed, proteomic analysis of diverse EV populations demonstrated that several common exosome markers, like major histocompatibility complex, flotillin, and heat-shock 70-kDa proteins, are actually present in all EVs 14. It has been thus suggested to use the generic term EVs instead of exosomes or microvesicles, possibly accompanied by an adjective describing EV properties in accordance to the isolation method 11. As an example, low ultracentrifugation speed isolates large EVs, while higher speed isolates small EVs. Peripheral blood carries a large number of heterogeneous EVs, which are currently explored for therapeutic and diagnostic purposes 15.

Experimental evidence have demonstrated the ability of EVs to shuttle and deliver a diversified range of molecules, including various types of nucleic acids and proteins 16,17, supporting the notion that EVs represent a novel route of paracrine/endocrine communication among different cells and even distant organs 18. The majority of studies regarding EVs in T2DM has posed the attention on microRNA (miRNA) cargo. miRNAs are small (22 nucleotides), highly conserved non-coding RNAs, that pair to sites within the 3' untranslated region of mRNAs, causing mRNA degradation and/or directly blocking translation into proteins 19. Increasing evidence suggests that miRNAs can be actively secreted outside the cell in association with EVs (or alternatively with protein complexes) and, being protected from degradation, can stably circulate in blood 20.

Accumulating data suggest that, during the development of T2DM and its CV complications, EVs are quantitatively and qualitatively altered 21,22,23. In particular, we here focus on the possible role of EV-shuttled miRNAs as both mediators and biomarkers of T2DM-dependent vascular complications, highlighting the emerging knowledge that may eventually develop into innovative nano-diagnostics and/or nano-therapeutics.

## Extracellular vesicles and the development of type 2 diabetes mellitus

### Mice and *in vitro* studies

While circulating EVs appear to derive from a large range of tissues 24, a seminal paper demonstrated that the adipose tissue represents the major source of circulating EV-shuttled miRNAs 25. Consistently, mice with an adipose -specific deletion of the miRNA-processing enzyme Dicer (ADicerKO) showed a marked decrease of circulating small EV-associated miRNAs, while the transplantation of adipose tissue (both white and brown) into ADicerKO mice was able to restore this alteration, and also promote an improvement of glucose tolerance. Of note, patients with lipodystrophy show decreased levels of circulating EV-miRNAs, overall supporting a framework where adipose tissue derived EV-miRNAs act as novel adipokines able to regulate gene expression in distant tissues 25.

The first manuscript demonstrating a link between horizontal transfer of miRNAs through EVs and T2DM showed that adipose tissue macrophages isolated from obese mice secrete miR-155-rich EVs which promote insulin resistance in both liver and skeletal muscles in naïve recipient mice, likely through suppression of PPARγ expression, a master regulator of adipose tissue function and glucose metabolism 26. Interestingly, the opposite was also shown since EVs from macrophages isolated from lean mice were able to improve insulin resistance in obese mice. However, both these findings were obtained by isolating EVs from *in vitro* cultured macrophages, thus allowing a “clean” and high-yield collection of EVs 26. These results were further substantiated and extended by a study showing that ultracentrifuge-isolated EVs from the bloodstream of obese, high-fat-diet (HFD)-treated glucose-intolerant mice were able to transfer this latter phenotype in otherwise untreated, lean mice 27. In this study, specific miRNAs were suggested to be involved, *i.e.* miR-122, miR-192, miR-27a-3p, and miR-27b-3p 25. Of note, this effect was obtained isolating a small amount of EVs from the circulation, supporting their relevant pathophysiological role in inducing insulin-resistance. These observations were replicated by loading specific miRNAs in “lean” EVs, *i.e.* miR-192, miR-122, miR-27a-3p, and miR-27b-3p, thus minimizing the risk that common EVs contaminants (*e.g.* pro-inflammatory lipids or proteins) may be the responsible for the observed effect. PPARα was again suggested as the main gene target of these EVs. Accordingly, lipid metabolism was also affected by EV treatment, since mice treated with EVs from both HFD-fed mice or loaded with miRNA mimic molecules induced a significant increase in the circulating levels of free fatty acids and triglycerides. Of note, these effects were also accompanied by an increase in adipose tissue inflammation and liver steatosis, overall recapitulating multiple key features of T2DM 27.

The above described three milestone manuscripts uncovered an additional possible mechanism underpinning the development of T2DM. However, it is still unknown whether the effect of EVs on metabolism is either transient or permanent and to what extent this mechanism can be exploited to reverse T2DM, rather than to induce it. In this context, the finding that EVs from normal, “lean” macrophages were able to attenuate insulin resistance has not been replicated using a small amount of EVs isolated from whole blood of a healthy animal, neither untreated nor manipulated 24.

Beside the central role of different PPAR isoforms, multiple potential mechanisms underpinning the development of insulin resistance have been shown *in vitro* using EVs isolated from commonly used T2DM models 21. EVs isolated from the adipose tissue of HFD-fed mice were reported to: 1- induce pro-inflammatory M1 macrophage polarization 28,29; 2- downregulate GLUT4 expression in adipocytes and skeletal muscle cells 30,31; 3- promote the cleavage of insulin receptor β-subunit in hepatocytes 32. Worth mentioning, both proteins and miRNAs seem to mediate these effects, since EVs are able to shuttle retinal binding protein 4 (RBP4), pro-inflammatory cytokines, miR-155 and other pro-inflammatory miRNAs, overall suggesting a coordinated propagation of low-grade inflammation (LGI) among different diabetes-relevant tissues. Notably, triggers related to overnutrition and aging are able to promote and sustain LGI, both locally in insulin-sensitive tissues and systemically. In turn, LGI is one of the main components driving insulin resistance, β-cell dysfunction, and other critical components of T2DM aetiopathogenesis 33. EVs are emerging as critical mediators of LGI in both T1 and T2DM 17 and also in autoimmune diseases 34. Another report showed that miR-155, miR-142-3p and miR-142-5p shuttled in lymphocyte-derived small EVs from prediabetic mice are able to promote the expression of MCP-1 and other chemokines when transferred to β-cells, thus accelerating β-cell deterioration 35. In addition, small EVs derived from bone marrow mesenchymal stem cells (MSC) isolated from aged mice promote insulin resistance in young mice, possibly through a miR-29b-3p mediated mechanism 36. Of note, the aging-associated pro-inflammatory drift, often referred to as “inflammaging” 33, is accompanied by a wide reshaping of the EV payload 37,17,38, which may explain, in part, the deleterious effect of the “old” microenvironment on a large range of diseases, including T2DM 39. Since the canonical anti-inflammatory treatments have shown limited success in preventing or attenuating T2DM development, EVs provide an attractive alternative to counteract LGI. Anti-cytokine therapies or other common anti-inflammatory compounds are able to slightly lower HbA1c but do not halt progression from prediabetes to frank T2DM 40,41, possibly due to a variety of reasons: the redundancy of pro-inflammatory pathways, the need of continuous treatment, and the lack of action on the actual molecular alterations promoting LGI. On the other hand, EVs transport complex packets of information composed of both proteins and nucleic acids, possibly reprogramming cellular responses in a long-lasting manner. At least four proof-of-principle evidences have been provided regarding the potential role of different EV preparations in attenuating multiple T2DM features. In particular: 1- EVs prepared from adipose tissue macrophages isolated from lean mice reverse insulin resistance of obese mice (with low-miR-155 content suggested as potential mediator of the benefit) 26; 2- EVs from adipose stem cells promote the differentiation of macrophages towards anti-inflammatory M2 phenotype (through STAT3-mediated induction of Arginase) 42; 3- EVs from bone marrow cells promote the proliferation of pancreatic β-cells (through miR-106b-5p and miR-222-3p-dependent down-regulation of the Cip/Kip pathway) 43; and 4- EVs from human MSCs ameliorate peripheral insulin resistance and limit β-cell destruction in T2DM rats 44. This latter finding is not surprising given the known immune suppressing properties of human MSC-derived EVs 45 and the lack of immunogenicity of EV transfer among different species 46. Indeed, EVs isolated from human MSCs are already being tested in clinical settings of autoimmune diseases 45. Thus, considering the pervasive role of LGI in the development and progression of T2DM, a therapy able to counteract the escalating, subclinical inflammatory responses might halt the progression of initial metabolic imbalances to manifest T2DM. Finally, given the increased easiness of manipulating EV content through electroporation or transient transfection (especially for small RNA) 47, artificial modification of patient-self EVs might also represent an attractive option. A slightly different approach, *i.e.* loading artificial nanoparticles with autoimmune-disease-relevant peptides bound to major histocompatibility complex class II (pMHCII), has already been proven efficient in reversing a number of autoimmune diseases, including T1DM, by triggering the generation and expansion of antigen-specific CD4+ T regulatory cell population 48. As mentioned above, β-cell dysfunction is a tangible phenomenon also in a subgroup of patients with T2DM 49.

Beside adipose, immune, and mesenchymal cell-derived EVs, also EVs derived from the pancreas, muscle cells, and the liver have been involved in some of the major features of T2DM. For instance, EVs released from hepatocytes exposed to lipotoxicity are enriched in miR-128-3p and are able to regulate the expression of PPAR-γ in recipient hepatic stellate cells, thus promoting a pro-fibrotic response 50. In addition, EVs derived from steatotic hepatocyte promote endothelial inflammation and facilitate atherogenesis by miR-1 delivery, KLF4 suppression and NF-κB activation 51. Hepatic EVs are also able to regulate energy metabolism in the adipose tissue. Indeed, it has been shown that small EV-derived miR-130a-3p could improve glucose intolerance via suppressing PHLPP2 to activate AKT-AS160-GLUT4 signalling pathway in adipocytes, overall preventing weight gain in mice exposed to a HFD 52.

Pancreatic islets have also been shown to secrete EVs enriched in mRNAs encoding key transcription factor and pancreatic hormones such as C-peptide and glucagon 53. Furthermore, immortalized mouse β -cell lines treated with pro-inflammatory cytokines secrete small EVs enriched in inflammatory miRNAs, *e.g.* miR-146a, promoting apoptosis in recipient β-cells or islets, thus propagating the damage triggered by LGI 54. On the other side, β-cells secrete also small EVs enriched in miR-26a that have been shown to ameliorate obesity-induced insulin resistance and hyperinsulinemia, an endocrine beneficial effect that is blunted in obese mice 55. Interestingly, the obesity-induced decrease of miR-26a within small EVs is reflected also in humans 55.

**Figure 1:**
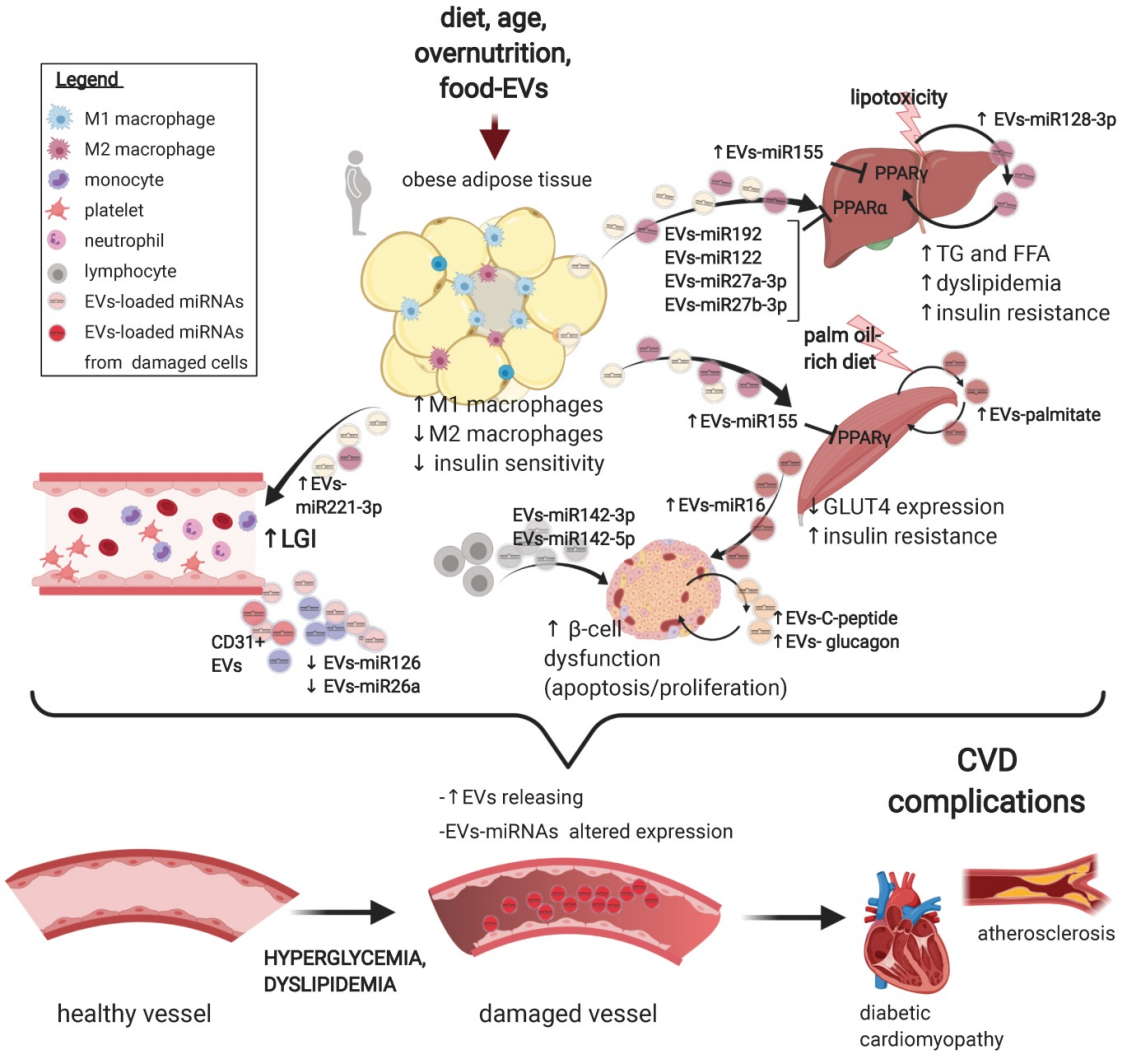
** Extracellular vesicles deregulation in the development of type 2 diabetes mellitus and its complications.** A schematic representation of the main preclinical findings regarding EVs alterations in the pathogenesis of T2DM and of its CV complications. A range of T2DM triggers promote a subclinical inflammatory response in the adipose tissue, altering the secretion and the loading of selected miRNAs onto EVs derived from resident macrophages. In turn, miR-155-rich EVs promote insulin resistance in liver and muscle by suppressing PPARγ expression. In addition, circulating EVs also shuttle a set of miRNAs, *e.g.* miR-122, miR-192, and miR-27a and b, which targets PPARα to induce hepatic insulin resistance, low-grade inflammation, and dyslipidaemia. Hepatocytes also secrete EVs rich in miR-128-3p in response to lipotoxaemia, promoting fibrosis in an autocrine manner. Muscle cells from mice subjected to a palm oil-rich diet release palmitate-abundant EVs which promote both muscle cells (autocrine signalling) and β-cells dysfunction in a endocrine manner. The vascular fraction of adipose tissue also secretes EVs rich in miR-221, able to directly target vascular smooth muscle cells. Insulin resistance, in turn, promote pancreatic β-cells dysfunction, an effect also independently sustained by lymphocytes-derived EVs abundant in miR-155, miR-142-3p and 5p. Finally, a wide range of T2DM-associated imbalances, *e.g.* hyperglycaemia and dyslipidaemia, foster an increased secretion of EVs of different origin with an altered payload, which, in turn, may negatively affect the progression of a plethora of CV complications of T2DM, including the progression of atherosclerosis and the development of diabetic cardiomyopathy. Figure created with Biorender.com

The skeletal muscle is key in regulating systemic insulin-sensitivity: skeletal muscle cells release both small and large EVs loaded with a wide range of molecules, which are currently being characterized for metabolic studies 56. A milestone study showed that small EVs isolated from muscles of mice treated with a palm oil-rich diet are enriched in palmitate and are able to alter the expressions of genes involved in cell cycle and muscle differentiation but alone are not sufficient to induce muscle insulin-resistance, as tested by Akt phosphorylation 57. A later study by the same group extended these findings, showing that mice skeletal muscle-derived EVs are also delivered to the pancreas *in vivo*. In addition, skeletal muscle-EVs derived from palm oil-treated mice induced proliferation in pancreatic islets, an effect likely mediated by a large derangement in the EV-miRNA payload and in particular by miR-16, suggested to target Ptch1 in β-cells and affect pancreatic development and function t 58. These findings are summarised in **Figure 1**.

Additional research is warranted to establish the magnitude and the duration of EV effect on LGI and metabolism and which molecules are responsible of the beneficial effect. In addition, translation of EV treatments (either as drugs or drug carriers) to clinical settings requires to solve a number of issues linked to the preparation of this novel class of biologicals, including the degree of purity, characterization and standardization among preparations. In this respect, the first guidelines on the topic suggest a series of key issues to further develop the field of EVs as nano-therapeutics 47. In addition, a deeper knowledge is needed to understand whether different EV subtypes produce different effects or whether the EV-payload, independently of the shuttle, is determining the observed phenotype.

### Human findings

Compared to animal experimentation, less information is available regarding the quantitative and qualitative alterations of EVs in human T2DM. The literature strongly suggests an increased number of either endothelial or platelet-derived EVs in patients with T2DM, with or without concomitant CVD 59. In particular, a meta-analysis evidenced that the number of large EVs from different sources, *i.e.* endothelium, platelets, monocytes, as well as total EVs are increased in patients with T2DM compared with controls 60. However, technologies able to indirectly quantify small EVs have only recently emerged. Thus, the majority of previous studies reported data only for large EVs, given the hurdle of conventional cytofluorimeter to reliably detect nanosized particles (unless they are further linked to larger beads) 61. A recent study has disentangled this issue by quantifying EVs with nanoparticle tracking analysis and specific EV-marker assays, showing that patients with T2DM have significantly higher levels of EVs in their circulation, in particular of erythrocyte origin, when compared to euglycemic controls 62. Insulin resistance was suggested as a putative trigger of the increased EV secretion. On the other hand, EVs from individuals with T2DM were preferentially internalized by circulating leukocytes, inducing a pro-inflammatory response *in vitro* 62. Similarly, patients with gestational diabetes also show an increased number of EVs which can induce inflammatory responses *in vitro* 63. Regarding quantitative alterations of EV-associated cargo, EVs from patients with T2DM showed a significant derangement of miRNA abundance. Among others (also discussed below), small EVs from patients with T2DM contain increased quantity of miR-20b-5p which in turn targets AKTIP and STAT3 to reduce insulin-stimulated glycogen accumulation 64. Another report showed that the quantitative dysregulation of several T2DM-affected EV-miRNAs are reversed by metformin treatment 65. Of note, the amount of miR-92a in small EVs isolated from human serum is correlated to brown fat activity, supporting the idea that EV-miRNAs are able to sense specific features of T2DM and thus express diagnostic potential^66^.

Human islets *in vitro* have been shown to secrete large EVs containing islet-specific proteins (insulin, C-peptide, GLP1R), the mRNAs encoding for VEGFa and eNOS, and miRNAs, *e.g.* miR-27b, miR-126, miR-130 and miR-296, involved in β-cell function, insulin secretion and angiogenesis. In turn, these EVs are able to induce insulin mRNA expression, protect from apoptosis and enhance the angiogenesis of recipient islet endothelial cells (EC)s 67. Worth mentioning, human islets have also been shown to secrete small EVs with specific miRNAs rearrangement kinetic when exposed to inflammatory cytokines. Indeed, EV-miR-29b-3p and 216a-5p can be detected early after the damaging stimulus and before any evidence of cell death, possibly providing a tool to detect islet damage before its appearance 68. On the contrary, miR-375-3p was enriched in EVs after 48 hours stimulation 68, a finding confirmed also in an independent study 69. Interestingly, EV-miR-375-3p was also elevated in patients with new-onset diabetes 69.

Overall, these data might support a diagnostic potential for EVs to detect early metabolic alterations or specific components of T2DM. While technology for EV isolation and characterization is progressing to ensure reproducibility among studies, the use of EV-shuttled miRNAs as efficient biomarkers is still hampered by the lack of a standardized method to normalize data. A number of approaches may be adopted to overcome this issue, *e.g.* the addition of non-human miRNAs before extraction, the use of a calibration curve with the miRNA of interest, or the use of digital PCR. In addition, further research may be guided by panels of experts developing consensus statements, similarly to what has happened with EV research (through the publication of commonly endorsed procedural guidelines by the International Society for Extracellular Vesicles, ISEV) 70.

## Extracellular vesicles and the cardiovascular complications of type 2 diabetes mellitus

### Mice and *in vitro* studies

EVs are being studied as putative, novel mediators of CVD. Accumulating evidence from animal models and cell systems suggest that EVs can propagate inflammation and worsen vascular damage *in vivo, ex-vivo* and *in vitro* 70. Several observations point to a functional passage of biological information *via* EVs among cells composing the blood vessels, and suggest the dysregulation of this phenomenon in diabetic conditions. EVs are particularly attractive as potential mediators of the residual vascular risk of patients with T2DM 71. As discussed above, aggressive reduction of CV risk factors reduces but does not suppress the incidence of CV events in T2DM. In addition, T2DM patients with no risk factors also have an increased risk of diabetic cardiomyopathy. These observations suggest that, beyond conventional risk factors, additional alterations concur to the genesis of T2DM complications.

A breakthrough manuscript showed that EV-mediated exchange of miR-126 supports endothelial growth, a mechanism that is blunted in T2DM conditions 72.* In vitro*, ECs exposed to high glucose release a higher number of EVs with an increased mean particle size, a greater pro-coagulant activity, and a higher potency to inhibit endothelial-dependent relaxation, supporting the hypothesis that such effects may actually contribute to progressive endothelial injury and subsequent CV complications in diabetes 73. Intriguingly, also senescence is able to modulate EV release and alter the miRNA payload of EC-derived EVs. In turn, these vesicles are enriched in miR-21-5p and miR-217 and are able to reduce cell growth, spreading a pro-senescence message 38. Of note, cellular senescence is induced by a variety of T2DM-related stressors 74,75.

EVs released by ECs in specific conditions can be up-taken by vascular pericytes, affecting the biology of these cells. As an example, high glucose promotes the shedding of endothelial EVs carrying miR‐503, whose transfer to vascular pericytes hampers their migration and proliferation 76. Large EVs derived from human coronary ECs and exposed to high glucose concentrations were also demonstrated to impair endothelial function, promoting increased macrophage infiltration and the expression of adhesion proteins in the atherosclerotic lesions of ApoE(-/-) mice, an effect mediated by an increased NADPH oxidase activity within these large EVs 77. Furthermore, EV-associated miR-92a released by ECs in response to atheroprone stimuli was demonstrated to suppress the expression of target gene KLF4 (Krüppel-like factor 4) in macrophages, suggesting a mechanism by which EV-associated miR-92a regulates the pro-inflammatory phenotypes of macrophage and, hence, atherosclerotic lesion formation 78, an effect observed also with large EVs from patients with coronary artery disease 79.

ECs are also targeted by EVs released by other cell types: it is the case of cardiomyocytes that, in diabetic rats, shuttle higher levels of miR-320 and lower amount of miR-126 and heat shock protein 20 (Hsp20) through small EVs compared to the same cells from non-diabetic rats. The transfer of these EVs to cardiac ECs results in a decreased expression of IGF-1, Hsp20, and Ets-2, thus impairing EC angiogenic function 80. Worth mentioning, these effects were reversed in a transgenic mouse model overexpressing Hsp20 in cardiomyocytes, likely due to an increased secretion of small EVs enriched with this protein 81. Garcia et al. have recently shown that small EVs derived from contractile cardiomyocytes can regulate glucose transport into the cardiac ECs. They have also reported that under hypoglycaemic conditions, cardiomyocytes produce a larger number of exosomes enriched with glucose transporters and enzymes involved in glucose metabolism, resulting in increased rate of glucose uptake and glycolysis in cardiac ECs under condition of glucose deprivation 82. It was found that blood circulating EVs from diabetic mice were easier to attach to the ECs and had more extracellular signal-regulated kinase (ERK)1/2 than did control mice-derived EVs, significantly altering endothelial function by activation of ERK1/2 pathway in these cells 83. Small EV-associated IgGs, whose quantity was found increased in diabetes, were observed to activate the classical complement pathway, demonstrating that these particles could contribute to the development of diabetic retinopathy; consistently, the lack of IgGs in exosomes in diabetic mice indeed resulted in a reduction of retinal vascular damage 84.

EVs isolated *ex-vivo* from perivascular adipose tissue of HFD-fed mice are enriched in miR-221-3p and can propagate low-grade inflammation in vascular smooth muscle cells (VSMC)s, promoting vascular dysfunction in femoral artery 85. Qualitative alterations within EVs have also been shown to promote pathogenic features of heart failure. Indeed, fibroblast-derived small EV-associated miR-21-3p can induce cardiomyocyte hypertrophy in a mouse model of Ang II-induced cardiac hypertrophy 86. Of note, both miR-21-3p and miR-21-5p have been reported to be affected by T2DM in various animal models 87. Furthermore, EV-associated miRNAs have been involved in diabetic nephropathy: in a mouse model of the early development of this complication, miR-145 was increased within the glomeruli and in urinary dispersed exosomes. Consistently, *in vitro* exposure of cultured mesangial cells to high glucose increased miR-145 content in both cells themselves and cell-derived exosomes, providing with a miRNA novel candidate player in diabetic nephropathy 88.

Notwithstanding the strong evidence that different EV sub-populations may display different properties and molecular cargos, only few studies have been posed to distinguish their distinct and specific biological function. An interesting paper showed that CD31+ EVs, but not whole-plasma EVs, are able to induce resistance to apoptosis in VSMCs, an effect mediated by membrane-bound platelet-derived growth factor-BB (mbPDGF-BB) and likely relevant for the progression of diabetic atherosclerosis 89. CD31+ EVs are held to mostly derive from ECs but also platelets and selected immune cells types express CD31, and, indeed, our unpublished observations support the heterogeneous nature of CD31+ EVs (authors unpublished data). Whether sub-groups of EVs, characterized by specific markers, are affecting specific types of cells, though, is still mostly unexplored. A possible alternative approach to study the effect of EVs from specific cell types is to collect them *in vitro* after isolation from primary samples. Through this approach, it has been demonstrated that treatment of mouse aortic rings with T cell line-derived EVs results in endothelial dysfunction in both conductance and resistance, though a decrease in expression of NO synthase and an overexpression of caveolin-1 in the endothelium 90.

Overall, a growing number of studies strongly suggests the involvement of EVs, and specifically EV-associated miRNAs, in the initiation and development of CV complications in T2DM. However, more studies are needed to establish the relevance of the collected observations, especially considering the large heterogeneity of the EV preparations used so far. In addition, there is still a limited knowledge regarding the target specificity of different EVs. On the other hand, the emerging picture supports the idea that EVs may represent an additional druggable target to prevent CVDs in patients with T2DM, especially for those complications which are only marginally affected by current multidimensional therapies, *e.g.* diabetic cardiomyopathy 91.

### Human findings

As mentioned above, EVs derived from patients with T2DM hold intrinsic pro-inflammatory properties, which might eventually contribute to fuel the pervasive status of LGI typical of the disease. It has been reported that EVs from patients with T2DM may shuttle higher levels of pro-inflammatory proteins, inducing cell lamellipodia formation and migration in recipient ECs *in vitro* 92. Similar findings were obtained also when exploring the content of pro-angiogenic factors within EVs from patients with T2DM, that was found significantly increased, especially in those patients with long disease duration and microvascular alterations 93. Consistently, a study exploring specific EV-markers in total EVs collected from a large cohort of patients with T2DM and manifest CVDs found that cystatin C levels in EVs were associated with prevalent metabolic syndrome while EV-CD14 levels were associated with a relative risk reduction for the development of T2DM 94.

T2DM has been shown to affect the abundance of a plethora of pro- and anti-angiogenic miRNAs (miR-193b-3p, miR-199a-3p, miR-20a-3p, miR-26b-5p, miR-30b-5p, miR-30c-5p, miR-374a-5p, miR-409-3p, and miR-95-3p). The quantitative dysregulation of these miRNAs associated to EVs from patients with T2DM compared to healthy controls suggested their involvement into the development of vascular complications due to impaired angiogenesis in such patients 95. In another study, circulating EVs isolated from subjects with T2DM were discovered to carry significantly reduced quantities of miR-126 and miR-26a (mostly released by ECs) compared to non-diabetic subjects and a more pronounced reduction of these miRNA quantity was associated with a higher risk for concomitant coronary artery disease. Consistently with clinical results, *in vitro* EC culturing experiments revealed that hyperglycemia reduces the packaging of miR-126 and miR-26a into EVs 96. Giving support to the idea that *in vivo* circulating EV function can be explored and dissected *in vitro*, plasma EVs from subjects with diabetic retinopathy have been demonstrated to induce features of retinopathy in *in vitro* models of retinal microvasculature, such as pericyte detachment and migration, and augmented permeability of pericyte/endothelial cell bilayers, when compared with EVs from controls 96. Similarly, also EVs secreted from pancreatic β-cells can enter the bloodstream and contribute to retinal injury, likely through an increased shuttling of miR-15a promoting oxidative stress 97. Of note, EV-shuttled miR-15a is negatively correlated with the thickness of the ganglion cell complex in patients with T2DM, possibly sensing initial stages of retinal damage 98.

It is relevant to highlight that miRNAs are not carried only by EVs but also circulate in blood associated with HDL or the RNA-binding protein argonaute-2 (Ago-2). Alteration of miRNA carrier distribution in plasma of patients with T2DM and diabetic nephropathy was reported in comparison with healthy control subjects, with carrier-specific miRNAs involved in endothelial barrier formation (EV-miR-21/126) and the pro-angiogenic response (HDL-miR-132) 99. Consistently with the mice experiments described above, miR-145 was found enriched in urinary exosomes from patients with incipient T1D-associated diabetic nephropathy, an observation that candidates miR-145 as a novel biomarker for this diabetic complication in the human setting 88.

These preliminary observations suggest that EV-miRNAs may mirror specific pathological mechanisms and also directly exert a functional role of complications development. Thus, EV-miRNAs might hold a greater diagnostic potential than whole-plasma miRNAs, which also result by the passive leakage of tissues and cell death 71 99. However, data regarding the diagnostic potential of EV-miRNAs to foresee long-term development of complications in prospective cohorts are still scarce. In addition, to be clinically meaningful, they should add diagnostic power to already available risk equations, often based on a limited number of variables routinely tested in clinical practice. Finally, the above-mentioned limitations regarding standardization of both EV collection and miRNA dosage also apply to the translation of EVs into novel nano-diagnostics for the complications of T2MD. To this respect, it should be stressed that differences due to various EV isolation procedures, RNA isolation techniques and sequencing platforms have been shown to affect the results of studies aimed at biomarkers discovery. The effort of The Extracellular RNA Communication Consortium (ERCC) is trying to disentangle all these aspects, including EV biogenesis and function, discovery of extracellular RNA biomarkers, development of RNA/EV-based therapeutics, and construction of a robust set of reference RNA profiles for a variety of biofluids 100, [http://exRNA.org/].

As evidenced here, a number of diabetes-related stressors, *e.g.* dysglycaemia and dyslipidaemia, modify the release and the payload of EVs from multiple tissues, and in particular their miRNA content. In turn, these altered EVs can eventually propagate an aberrant signature that modifies the epigenetic set‐up in receiving cells even after risk factors-reduction. This would perpetuate the insult despite glucose and lipid normalization, a phenomenon that might contribute to the residual vascular risk of patients with T2DM 56. The recognition of EV pathogenic involvement in diabetic CV complications may significantly support the development of EV-based therapeutics; the identification of both cell source and destination of EVs and the understanding of EV organ-tropism may uncover specific cell-to-cell communication modules and help the design of EV-based therapies to target specific tissues and/or cells. In particular, we will need to comprehend how the chronic inflammatory environment of T2DM affects cargo packaging and release of EVs and, in turn, what are the consequences at the systemic level of this EV dysregulation. Possible therapeutic approaches are based on either reversing pathological effects of EV-associated miRNAs by the use of antagomir molecules or activating miRNA-dependent protection by the use of miRNA mimics 114. EVs have the potential to be engineered not only with the addition of a cargo molecule, but also with surface proteins able to redirect the EVs to specific EV target cells, thus reducing unwanted off-target effects 115.

While several obstacles and uncertainties keep hampering the development of EV-based therapeutic strategies for the treatment of CV diseases, encouraging results in animal models showing the capability to prevent fibrosis, cardiac hypertrophy and inflammation candidate these biological particles as promising nano-therapeutics.

## Effect of different nutrients and dietary regimens on extracellular vesicles

Diet is a crucial risk factor for the development of T2DM and its complications 101 102. Few studies have assessed the effect of different dietetic approaches on the modulation of EVs, as well as the effect of specific nutrients on EV release and cargo *in vitro*.

### *In vitro* studies

As mentioned above, high glucose can increase the release of EVs by ECs and trophoblast cells *in vitro*, while hypoglycaemia can trigger EV release by cardiomyocytes and affect cargo composition 73 82 103. In turn, these EVs are able to spread inflammation when administered to recipient cells 103. Similarly, the saturated fatty acid palmitic acid increases the number of secreted EVs in both hepatocytes and proximal tubular epithelial cells 104,105. Of note, EVs derived from palmitic acid-stimulated hepatocytes promote the expression of fibrotic genes in hepatic stellate cells 104. Both high glucose and palmitic acid, two of the most used *in vitro* models of overnutrition, induce an increase in EV secretion. Paradoxically, nutrient deprivation also increases the secretion of small EVs without affecting their cargo composition, an effect mediated by mTORC1 inhibition and phenocopied by rapamycin 106. These apparently contrasting findings may be explained by the diversity of the models used and the heterogeneity of the EV populations analysed. Indeed, small EVs often derive from multivesicular bodies while larger EVs mainly derive from plasma membrane, with different pathways controlling their production and secretion 107. Finally, the olive oil polyphenol hydroxytyrosol (HT) is able to prevent the TNF-α-induced upregulation of exosome-loaded miR-34a and miR-155 in adipocyte and monocyte cell lines and inhibit the NF-kB pathway and ROS production 108, providing a proof-of-principles that non-energetic constituents of food may also affect EV biology *in vitro*.

### *In vivo* human findings (effects of diet and exercise)

A milestone study suggested that a 4 week Mediterranean diet enriched in extra virgin olive oil is effective in reducing the shedding of CD31+ and CD144+/CD62E+ EVs from ECs in elderly subjects at moderate-to-high CV risk 109. On the contrary, a highly saturated fat meal resembling a fast-food diet leads to increased platelet and CD144+ endothelial EV release in healthy individuals 110. Patients affected by coronary artery disease subjected to one-month daily supplementation with 375 mg of cocoa flavonols displayed a decreased endothelial CD31+/CD41- and CD144+ EV shedding. Similarly, the same effect was observed with 13 grams per day of cocoa powder in overweight or obese, but not in normal-weight women 111. In a recent cross-sectional study, elderly patients with a prior acute myocardial infarction (AMI) were administered a Nordic diet, which is rich in long-chain omega 3 fatty acids, fiber, minerals, and antioxidants, to test its effect on peripheral blood circulating EVs. A major adherence to the Nordic diet was associated with a lower percentage of total Annexin V (AV)+ and platelet-derived (CD61+/AV+ and CD31+/AV+) EVs in AMI patients 112, an effect observed also with the Mediterranean diet 113. Finally, in one of the few studies conducted with T2DM patients, an 8 week oat-enriched diet has shown to reduce platelet- and monocyte-derived large EVs relative to a standard diet 114. Overall, these findings may support a framework where a high intake of saturated fatty acid induces an increment of platelet- and EC-derived EV shedding, while a high intake of omega-3, polyphenols, fibers and polyphenol-rich products are associated with a reduction in the circulating number of several EV populations.

Physical exercise is known to induce a plethora of beneficial metabolic effects. The most powerful approach to halt the progression of prediabetes to manifest T2DM is an intensive lifestyle modification introducing at least 150 minutes of physical activity per week 115. While the calories-consuming and myokines-releasing properties of physical exercise are well studied, an effect mediated by EVs has also been proposed 116. In particular, physical exercise induces a rapid release of small EVs into the circulation, an effect that starts in the aerobic phase of exercise and is further promoted by lactic acid accumulation 117. As expected, some of the EVs released during acute physical activity derive from the skeletal muscles, as evidenced by a large positivity for alpha-sarcoglycan 118, but also platelet, endothelial, and immune cells have been reported to contribute to the released EV pool 24. A recent study has analysed the proteome of EVs released during acute exercise, revealing that 35 candidate myokines are released within muscle-derived EVs and functionally shuttled to the liver 119.

To our knowledge, no study has ever tested whether EVs derived from trained animals or during acute exercise is able to attenuate hyperglycaemia or any features of T2DM in a sedentary recipient. However, studies conducted thus far support the assumption that EVs might be considered as novel myokines, possibly mediating part of the beneficial effect of physical exercise. If this hypothesis is confirmed, EV parabiosis (transfer from one organism to another) from trained athletes to patients with T2DM might hold potential to ameliorate the progression of the disease.

### Food-contained, EV-shuttled miRNAs

Beside the indirect effect in mediating EV biology, recent works suggest that food itself contain EVs. Evidence for EV presence have been found in a wide range of dietary sources, of both animal and vegetal origins 120,121. Plants have adapted EV-mediated cross-kingdom RNA interference as part of the immune responses against pathogens 122, an effect suggested to be active also against mammalian infection 123. More broadly, intra- and/or inter-species cell-cell communication through EVs is emerging as a conserved mechanism exploited by almost all living beings 124. However, whether EVs contained in food are biologically active is matter of debate 125.

Milk is the most studied food source to decipher the functional roles of EVs, particularly focusing on their miRNA content. A large fraction of miRNAs in milk is loaded into EVs and more than 400 miRNAs have been identified in bovine milk, the vast majority of which share identical nucleotide sequences with humans, and thus have the potential to regulate human genes 126. As a proof-of principle of their bioactivity, two EV-loaded miRNAs rich in cow milk, miR-29b, and miR-200c, were found upregulated in human plasma till four to six hours following milk consumption 127,128. The high content of EV-shuttled miR-29b has been proposed to promote the development of T2DM, due to its role in the activation of mTORC1-mediated insulin resistance and SPARC-mediated dysfunction in insulin-secretion 126. Similarly, milk EVs contain high quantities of miR-21 and miR-148a, which might eventually accelerate the development of atherosclerosis through the induction of macrophage M1 polarization and the repression of LDL receptor 126.

**Figure 2:**
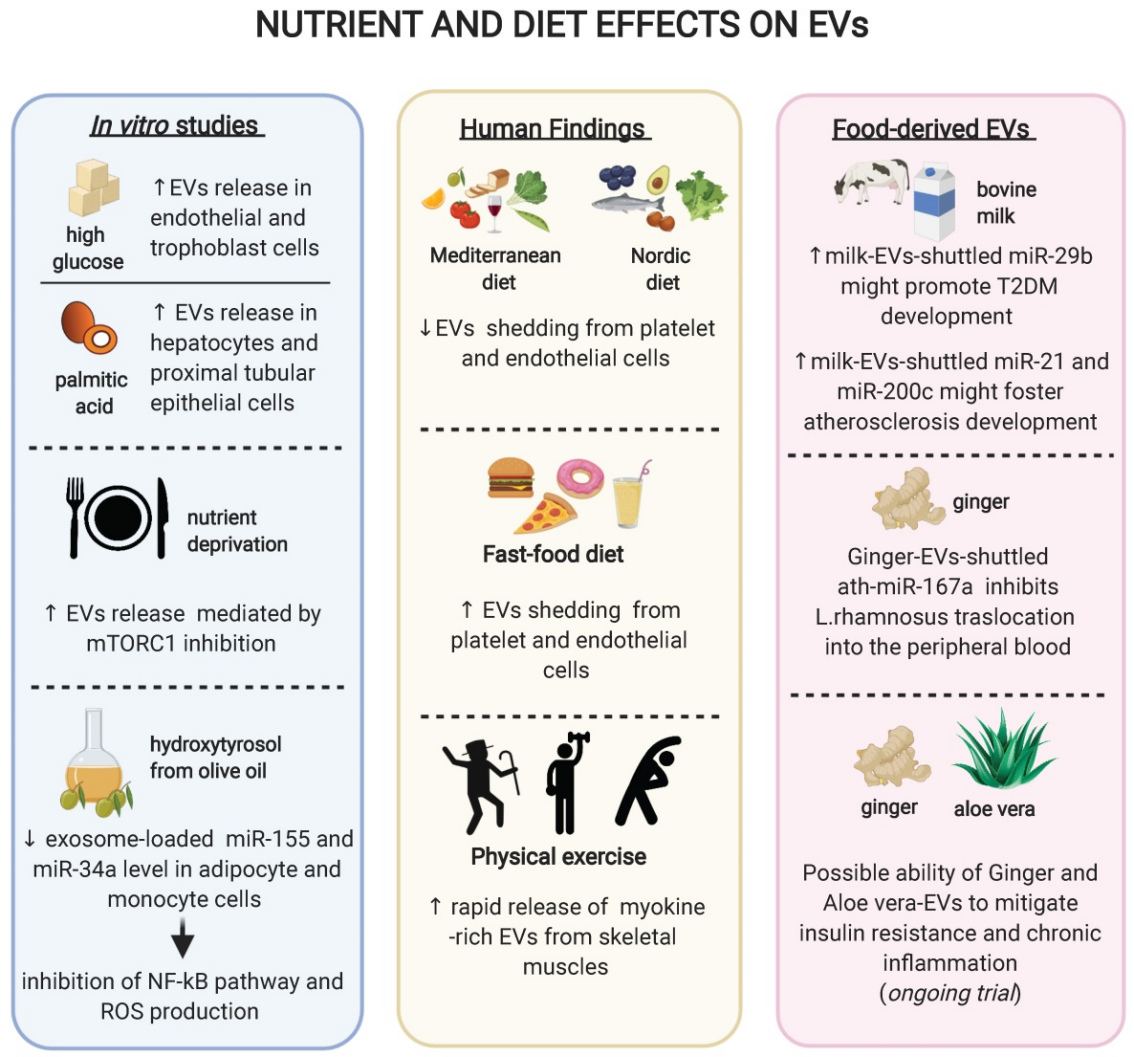
** Schematic representation of the effects of diet, exercise or different nutrients on extracellular vesicles.** *In vitro* studies revealed that different nutrients might modulate the EVs release and/or their content (*box on the left*); clinical studies have reported that diet as well as physical activity might influence the shedding of EVs from various tissues (*box in the middle*); novel findings on food-contained EVs showed possible different roles of EV-loaded miRNAs on human pathology (*box on the right*).

Large cohort studies suggested that the prevalence and incidence of T2DM are significantly lower among individuals following plant-based eating patterns compared with conventional diets 129. Interestingly, while a lower BMI has been suggested to mediate the beneficial effect of this diet, in some cases the differences in diabetes risk persist after adjustments for adiposity 130. Plant-derived food has also been shown to contain EVs and in particular EV-shuttled miRNAs. In particular, 418 conserved miRNAs were identified from 11 edible fruits and vegetables such as ginger and soybean 131,132. A breakthrough manuscript suggested that EVs contained in ginger is able to shape the gut microbiota. In a study conducted on a mouse model of colitis, the ginger EV-derived miRNA ath-miR167a could directly bind the Lactobacillus rhamnosus pilus SpaC mRNA, thus blocking SPAC protein expression. As a result, Lactobacillus rhamnosus showed a reduced translocation into the peripheral blood accompanied by a diminished bloodstream infection 112. Of note, the translocation of detrimental bacterial species (or derived molecules, *e.g.* pro-inflammatory LPS) from gut leakage to the liver is held to be one of the main mechanisms linking dysbiosis to the development of hepatic insulin resistance and possibly obesity-induced T2DM 133 3. The effect of diet and exercise on circulating EVs is summarised in **Figure 2**.

Currently, few randomized clinical trials exist that evaluate the effect of food intake on EV shedding form different cell populations involved in CV complications, while no trial data have been published regarding the clinical effect of food-derived EVs. One ongoing trial is testing the ability of plant-derived EVs (*i.e.* ginger and aloe) to mitigate insulin resistance and chronic inflammation in patients with polycystic ovary syndrome and will likely provide with the first preliminary cues about the potential of this approach to translate into T2DM settings (NCT03493984). As a matter of fact, more research is needed to explore the bioavailability, distribution and actual potential of food-derived EVs in humans. However, such an approach could eventually open new opportunities regarding the employment of plant-derived EVs as active drugs themselves or for the delivery of therapeutic agents, given the potential to prepare large amount of biocompatible EVs.

## Conclusions and future prospects

EV biology is a rapidly growing field of research, with the potential to revolutionize the diagnosis and the therapy of a wide range of complex, multi-factorial diseases, including T2DM and its CV complications. EV dysregulation has been involved in virtually all stages of the T2DM trajectory, affecting the development of insulin resistance, β-cell dysfunction, dyslipidaemia, and atherosclerosis, as well as of other CV complications (**Figure 1**). However, additional research is needed to clarify the effective contribution of EVs to pathological processes relevant for T2DM development and progression in humans. Indeed, human findings collected thus far are associative rather than causative. Also, standardized methods for isolation, preparation, characterization, and quantitation of EVs are mandatory to progress to clinical stages, for both diagnostic and therapeutic purposes. EV number and molecular cargo have also been shown to be affected by diet and exercise, two of the most powerful strategies to prevent T2DM and CVDs. This picture is further puzzled by the observations that food itself might contain biologically active EVs (resumed in **Figure 2**). Given the progressively increasing easiness of their manipulation, EVs from multiple sources may represent the ideal candidates to eventually improve a number of aspects of T2DM treatment and management, such as attenuation of insulin resistance in initial stages, amelioration of β-cell failure, improved risk stratification for CVD development, and modulation of pathways promoting the development of complications. Considering the complex package of information shuttled, EVs may eventually affect these pathological components in a long-term manner, providing an additional and different strategy to conventional therapies. On the other hand, it is unlikely that such a powerful tool would not be accompanied by side effects, bearing in mind that EV-shuttled molecules, including miRNAs, are known to display a pleiotropic activity. Thus, while pre-clinical research is still in its infancy, we can foresee that the translation to a clinical stage will require tailored and specific settings. However, available evidence encourages further research to explore the potential of EVs as future nano-diagnostic and nano-therapeutic tools for T2DM management.
